# Fournier's gangrene in a patient after third-degree burns: a case report

**DOI:** 10.1186/1752-1947-3-7264

**Published:** 2009-05-26

**Authors:** Christos Iavazzo, Konstantinos Kalmantis, Vasiliki Anastasiadou, George Mantzaris, Vallantis Koumpis, Fotinie Ntziora

**Affiliations:** 1 Surgical Department, Vougiouklakeion Hospital, Athens, Greece; 2 Internal Medicine Department, Vougiouklakeion Hospital, Athens, Greece

## Abstract

**Introduction:**

Fournier's gangrene is characterized by tissue ischemia leading to rapidly progressing necrotizing fasciitis.

**Case presentation:**

We present the case of a patient with Fournier's gangrene after third-degree burns. Clinical manifestations, laboratory results and treatment options are discussed.

**Conclusion:**

Fournier's gangrene is a surgical emergency. Although it can be lethal, it is still a challenging situation in the field of surgical infections.

## Introduction

Fournier's gangrene is a rare clinical entity which usually presents in debilitated patients with comorbidity and systematic disorders such as diabetes mellitus, alcoholism, immunosuppression and perianal infection. The epidemiology of Fournier's gangrene has changed from its original description. Currently, the disease is present in both genders, affects a wide range of ages and has a more insidious onset than in the past. It has been suggested that poor socioeconomic conditions could contribute to the development of Fournier's gangrene. Better understanding of the pathophysiology has reduced the ratio of idiopathic cases.

We present the case of a patient with Fournier's gangrene following third-degree burns. The aim of our study is to point out that Fournier's gangrene is a surgical emergency with a high rate of mortality, regardless of the survival benefits accumulating from recent advances in health care.

## Case presentation

**Figure 1:**
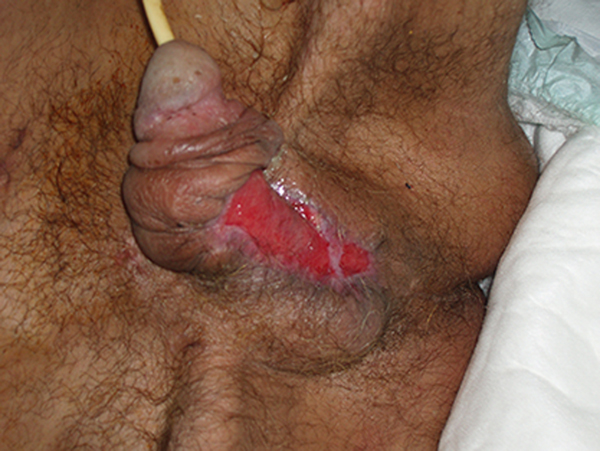
**Fournier's gangrene**. Swelling lesions on the scrotum and penis with edematous and irregular ulcer edges.

A 65-year-old man presented at the emergencies department of our hospital with third-degree burns due to a recent suicide attempt. On arrival, the patient reported scrotal pain; swelling lesions could be seen on the scrotum and penis (Figure 1). High fever (up to 39°C) was also present. Clinical examination revealed a 4 × 5 cm ulcerated lesion on the penis and scrotum. The edges of the ulcer were edematous and irregular. The surrounding tissues were rather necrotic. A brown, seropurulent, exudative, and mousy odor was characteristic. Decolorization of the skin was also found and the wound invasion was rather quickly increasing. Fournier's gangrene was diagnosed. Comorbidity included diabetes mellitus known for the last 5 years and major depression known for the last 25 years.

Microbiological testing returned: Hct: 31.7%, Hb: 11.2 g/dl, WBC: 26,3 K/μl (neut/lymph: 92.4%/4%), PLT: 326 K/μl, urea: 106 mg/dl, creatinine: 1.7 mg/dl, Na: 139 mMol/L, K: 4.1 mMol/L, Leu: 6 g/dl, Alb: 2.2 g/dl. A computed tomography (CT) scan revealed that the liver and spleen were enlarged and that the prostate gland showed benign hypertrophy. Blood and urine cultures were all negative. However, cultures of the necrotizing tissues grew *Escherichia coli*. This was the reason why the differential diagnosis included an infected burn. However, clinical signs and symptoms were characteristic of Fournier's necrotizing fasciitis.

The patient was treated with ciprofloxacin twice daily (intravenous) according to the antibiogram. Human albumin twice daily was also added and furthermore a protein-rich diet was also initiated. Careful daily inspections of the wound were necessary to determine whether the lesions were viable or necrotic. Aggressive debridement of the wound was followed by twice daily dressings with NaCl 15% solution and betadine solution. No skin graft was used. Our patient was also treated for diabetes mellitus and depression.

On follow-up 2 months later, a significant improvement in the wound was noted. However, the patient presented with diarrhea and anemia with hematocrit down to 28%. Stool examination revealed medium blood loss (Mayer test ++). Colonoscopy followed the diagnostic aspect and revealed two polyps in the sigmoid. Polypectomy was performed with an electrocautery snare during the colonoscopy. The histological diagnosis was tubular adenoma. Six months after first diagnosis, the condition of the patient had improved, characterized by healing of burns and improvement in Fournier's gangrene as the lesions were no longer necrotic but viable. A skin graft is planned for better healing.

## Discussion

Fournier's gangrene or necrotizing fasciitis of the perineal, perianal or genital regions is a challenging situation in the field of surgical infections. Tissue ischemia is the main pathogenetic factor and it is usually characterized by rapidly progressing myonecrosis and/or necrotizing fasciitis, leading to thrombotic occlusion of small subcutaneous vessels and development of gangrene. It is usually caused by polymicrobial infection, both aerobic and anaerobic bacteria [1]. The most commonly isolated microbe is *Escherichia coli* - as in our patient - followed by *Streptococcus, Staphylococcus* species, *Enterobacter cloacae, Enterococcus faecalis* and *Klebsiella pneumonia*[2]. Although broad-spectrum antibiotic prophylaxis is used and modern operating techniques are performed, the mortality rate is still high reaching 14.7% in non-diabetic and 33% in diabetic patients [3].

A Fournier's Gangrene Severity Index (FGSI) was created by Laor *et al.* in 1995 by modifying the acute physiology and chronic health evaluation (APACHE) II severity score [4]. In the FGSI, nine parameters are measured and the degree of deviation from normal is graded from 0 to 4. Parameters examined include temperature, heart rate, respiratory rate, serum sodium, potassium, creatinine and bicarbonate levels, hematocrit and leukocyte count. Regression analysis among different studies has shown a strong correlation between the FGSI score and the death rate.

In a recent study by Fajdic *et al.* including seven male patients with mean age 61 years ranging from 57 up to 66 years, it was shown that diabetes mellitus, urethrostenosis, hemorrhoids, anal fissure and abscesses might be strongly correlated with Fournier's gangrene [5]. According to a study by Unalp *et al.,* Fournier's Gangrene Severity Index (FGSI) > 9, diabetes mellitus and sepsis on admission were found to be factors for an unfavorable prognosis [6]. Chronic renal failure, hepatic failure, prosthetic penile implants, AIDS, malignancy and obesity were also important risk factors. Fournier's gangrene has been described in immunosuppressed patients following liver, renal or even cord blood stem cell transplantation [4,5]. We should note that Fournier's gangrene may represent the sole sign of underlying malignancy, as was reported in a Romanian study where such a case was the unique sign of a lower rectal adenocarcinoma [7].

Fournier's gangrene may still have an idiopathic origin that usually leads to a refractory situation [8,9]. It should be mentioned that our patient was a 65-year-old man with diabetes and anemia and with benign polyps of the sigmoid colon.

Fajdic *et al.* suggested that treatment has the potential to be successful when it is started at the onset of the disease and is aggressive, such as with necrectomy and broad antibiotic protection [5]. The therapeutic role of locally 100% oxygen in daily doses is also discussed [10]. Hyperbaric oxygen therapy may be a useful adjunct, but it is not a substitute for surgery and, consequently, it must not be allowed to delay the surgical debridement of an invasive soft tissue infection. Reconstruction of defects can also be offered by using local skin flaps [11]. Colostomy, urinary diversion or orchiectomy have also been suggested but have only been used for extensive and complicated cases [12].

## Conclusion

Fournier's gangrene exists and can still be lethal. It is a rare condition in Europe. Furthermore, such a complication of burns must be diagnosed early or even prevented. A high index of clinical suspicion is necessary before the local signs indicate detrimental fasciitis.

## Consent

Written informed consent was obtained from the patient for publication of this case report and any accompanying images. A copy of the written consent is available for review by the Editor-in-Chief of this journal.

## Competing interests

The authors declare that they have no competing interests.

## Authors' contributions

CI was the author, KK and FN were co-authors and contributed equally in writing the paper with CI and were the major contributors, VA and FN have been involved in drafting the manuscript and revising it critically, VK and GM have analyzed and interpreted the patient data. All authors read and approved the final manuscript and took public responsibility for the appropriateness of the content.
